# Solid-State Nuclear Magnetic Resonance of Triple-Cation Mixed-Halide Perovskites

**DOI:** 10.1021/acs.jpclett.2c02313

**Published:** 2022-10-06

**Authors:** Noemi Landi, Elena Maurina, Daniela Marongiu, Angelica Simbula, Silvia Borsacchi, Lucia Calucci, Michele Saba, Elisa Carignani, Marco Geppi

**Affiliations:** † Department of Chemistry and Industrial Chemistry, University of Pisa, via G. Moruzzi 13, 56124Pisa, Italy; ‡ Department of Physics, University of Cagliari, S.P. Monserrato-Sestu Km. 0700, 09042Monserrato, Cagliari, Italy; § Institute for the Chemistry of OrganoMetallic Compounds - ICCOM, Italian National Research Council - CNR, via G. Moruzzi 1, 56124Pisa, Italy; ∥ Center for Instrument Sharing, University of Pisa (CISUP), 56126Pisa, Italy

## Abstract

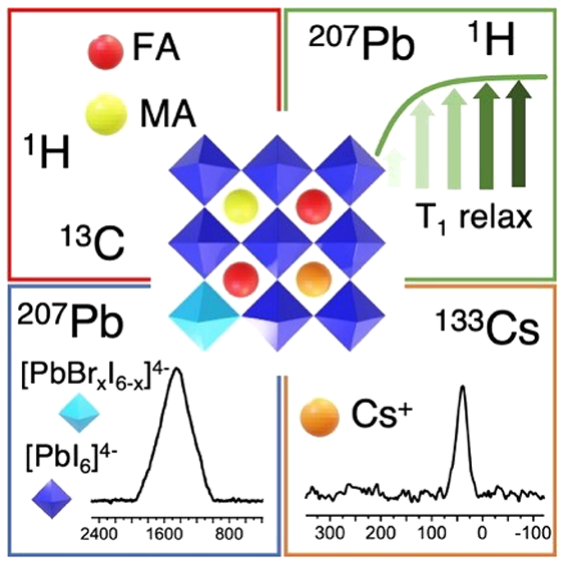

Mixed-cation lead mixed-halide perovskites are the best candidates for perovskite-based photovoltaics, thanks to their higher efficiency and stability compared to the single-cation single-halide parent compounds. TripleMix (Cs_0.05_MA_0.14_FA_0.81_PbI_2.55_Br_0.45_ with FA = formamidinium and MA = methylammonium) is one of the most efficient and stable mixed perovskites for single-junction solar cells. The microscopic reasons why triple-cation perovskites perform so well are still under debate. In this work, we investigated the structure and dynamics of TripleMix by exploiting multinuclear solid-state nuclear magnetic resonance (SSNMR), which can provide this information at a level of detail not accessible by other techniques. ^133^Cs, ^13^C, ^1^H, and ^207^Pb SSNMR spectra confirmed the inclusion of all ions in the perovskite, without phase segregation. Complementary measurements showed a peculiar longitudinal relaxation behavior for the ^1^H and ^207^Pb nuclei in TripleMix with respect to single-cation single-halide perovskites, suggesting slower dynamics of both organic cations and halide anions, possibly related to the high photovoltaic performances.

Hybrid organic–inorganic perovskites are very promising energy materials first reported as active layers in solar cells in 2009^1^ and now employed in solar cells exceeding 25% efficiency and even approaching 30% in tandem configuration with silicon.^2^ Perovskites have general formula ABX_3_, and the ideal crystal structure, often distorted, consists of a cube defined by eight corner-sharing [BX_6_]^4–^ octahedra, with the A^+^ cation sitting in the center of the cube (Figure 1a). Hybrid lead halide perovskites have an organic cation (or a mix of organic cations) and Pb^2+^ as A^+^ and B^2+^ cations, respectively, and the halide (X^–^) can be Cl^–^, Br^–^, I^–^.

**Figure 1:**
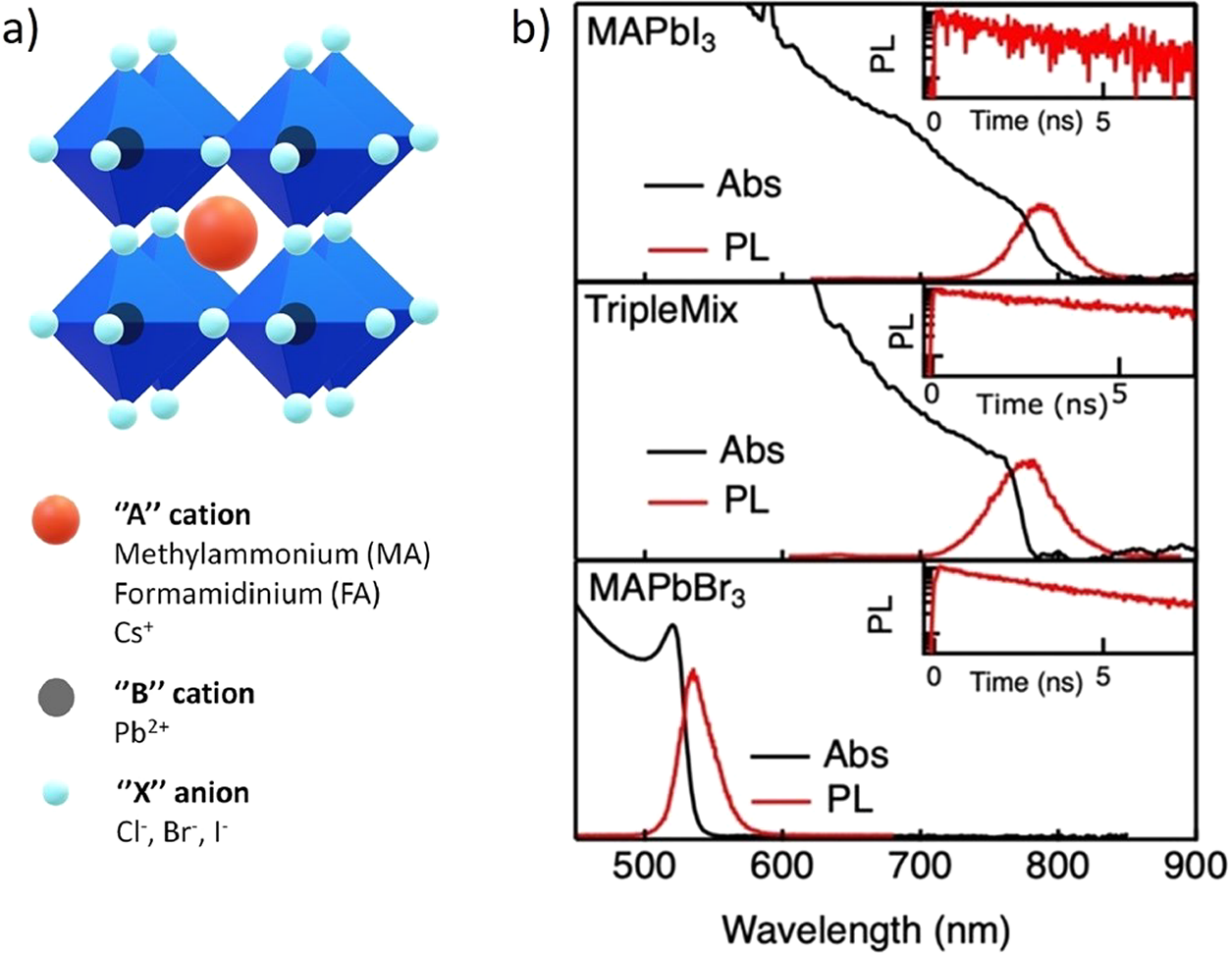
(a) Representation of cubic perovskite structure: eight corner-sharing [BX_6_]^4–^ octahedra (blue) forming a cube at the center of which the A^+^ cation (orange) lies. (b) Absorption and photoluminescence spectra of MAPbI_3_, TripleMix, and MAPbBr_3_. (insets) Photoluminescence decays with time.

Attractive features of perovskites include high optical absorption coefficients, long carrier diffusion lengths and lifetimes, small exciton binding energies, high photovoltaic performance, and, especially, the possibility of tuning their optoelectronic properties by changing the stoichiometry and microstructure.^3^ Over the past decade, Perovskite Solar Cells (PSCs) have reached very high Power Conversion Efficiency (PCE), comparable to that of silicon-based ones.^2,4^ However, issues, arising from structural instabilities and/or the inherent instability in air of the organic cations present in hybrid perovskites, hinder practical applications and commercialization of PSCs.^5,6^ Despite their limitations, metal-halide perovskites are considered exceptional candidates for photovoltaic (PV) applications. Indeed, there is currently substantial interest and ongoing research efforts in developing stabilization strategies for PSCs. In the past few years, it has been found that such stabilization can be achieved, for example, by including different A-site cations and mixing halides into perovskite compositions. To date, perovskites containing a mixture of formamidinium (FA), methylammonium (MA), and cesium (Cs^+^) as monovalent cations and a mixture of bromide and iodide as anions are among the best-performing PSCs.^7−9^

From a structural point of view, a change in the shape and relative orientation of the [BX_6_]^4–^ octahedra (Figure 1a) can lead to modifications of stability, polymorphism, and optical properties of perovskites. The Goldschmidt tolerance factor^10^ (*t*) can be used to predict which phase will form on the basis of the ionic radii of the A^+^, B^2+^, and X^–^ ions (). Perovskites with a tolerance factor of 0.9–1.0 have an ideal cubic structure, while *t* values between 0.71 and 0.9 result in a distorted perovskite structure with tilted octahedra.^11^ The only A-site cations that are known to allow the formation of cubic perovskite structures are MA, FA, and Cs^+^. Na^+^, K^+^, and Rb^+^ cations are too small, and organic cations such as ethylaminium, ethylenediaminium, and guanidinium are too large to form a cubic structure on their own, but they can all be used as additives for phase stabilization and defects passivation.^12^ In fact, even though MA, FA, and Cs^+^ have the ability to form cubic structures, pure MAPbI_3_, FAPbI_3_, and CsPbI_3_ present some limitations to their practical use in solar devices: (i) MAPbI_3_ decomposes into methylammonium iodide and PbI_2_ under external stimuli (e.g., humidity, temperature);^13,14^ (ii) FAPbI_3_ has a lower band gap than MAPbI_3_, resulting in a higher efficiency, but its photoactive perovskite α-phase (“black phase”, α-FAPbI_3_) transforms into the undesirable photoinactive, nonperovskite δ-phase (“yellow phase”, δ-FAPbI_3_) at ambient conditions;^15,16^ (iii) CsPbI_3_ is stable in the desired photoactive cubic phase only at temperatures above 300 °C, while at room temperature it converts into the photoinactive δ-phase (δ-CsPbI_3_).^7^ The perovskite phases of CsPbI_3_ and FAPbI_3_ are structurally unstable because Cs^+^ and FA cations are, respectively, smaller and larger than MA and do not optimally occupy the A-site.^17^

Compositional engineering strategies have been extensively explored to overcome the stability issues while pushing the efficiencies higher and higher. By mixing MA and FA cations, better PV performances can be obtained:^18,19^ the addition of MA stabilizes the photoactive phase of FAPbI_3_, and the charge transport properties improve.^20^ However, even in the presence of MA, traces of the yellow phase may still form, affecting the crystal morphology and, consequently, the performance.^7^ Moreover, it has been suggested that substituting a small amount of I^–^ with the smaller Br^–^ would improve the perovskite stability.^21,22^ However, I^–^ and Br^–^ mixing can result in a partial phase segregation when their amounts are nearly equivalent, and this is detrimental to the PSC performance and stability; therefore, attention must be paid to the I^–^/Br^–^ ratio.^3^

Saliba and co-workers added Cs^+^ as a stabilizer to MA/FA mixed perovskites and developed the first solar cells based on a Cs^+^/MA/FA mixed cation perovskite with mixed halides, obtaining high performances and improved stability.^7,23^ In fact, even a small amount of Cs^+^ is sufficient to effectively suppress the yellow phase of FAPbI_3_ and induce the formation of highly uniform perovskite grains. The beneficial effects of the addition of Cs^+^ to perovskites were investigated and demonstrated also by Zhang et al.^24^

As mentioned, perovskite compositional flexibility is ideal to tune materials’ properties. However, understanding the relationships between composition, properties, and performance is still a challenge. Given the complexity and the broad compositional and structural variety of these materials, this issue can only be addressed by a multidisciplinary approach. In particular, in addition to functionality studies (e.g., studies focused on the synthesis routes and the measurement of optical properties), detailed characterizations of the perovskite structural features and ion dynamics are essential. Techniques typically used to study perovskites are mostly X-ray/electron/neutron diffraction, UV–vis absorption, and photoluminescence (PL) spectroscopies. Recently, it emerged that crucial information on these compounds can be obtained by means of Solid-State Nuclear Magnetic Resonance (SSNMR) spectroscopy, which proved to be very effective in highlighting aspects such as cation incorporation, phase segregation, halide mixing, decomposition mechanisms, disorder, and dynamics, as summarized in recent reviews.^25−29^ The advantage of SSNMR substantially arises from the possibility of observing many different NMR-active isotopes, each having several spin properties sensitive to the atom environment as well as to interactions and dynamics.

SSNMR has proven to be extremely useful for the study of mixed perovskites. For instance, it has been employed to investigate halogens mixing in solid solutions of Cl/Br and Br/I Lead Halide Perovskites (LHPs) of general formula MAPbX_3_^30^ and FAPbX_3_,^31^ focusing on the miscibility, phase segregation, and local environments of Pb nuclei. Moreover, the effect of A-cations mixing has been studied by SSNMR in MA_*x*_FA_1–*x*_PbI_3_^32,33^ and Cs_*x*_FA_1–*x*_PbBr_3_^34^ double-cation mixed perovskites. In addition to phase segregation, the dynamics of FA and MA was investigated in the latter samples. While no differences in the dynamic behavior were found in MA_*x*_FA_1–*x*_PbI_3_ with respect to the parent pure perovskites, in the case of Cs_*x*_FA_1–*x*_PbBr_3_ the FA dynamics and phase transitions were affected by the presence of Cs^+^. Increasing the complexity of the systems, the incorporation of Cs^+^, Rb^+^, and K^+^ in MA_*x*_FA_1–*x*_PbI_3_ was investigated by SSNMR in double-, triple-, and quadrupole-cation perovskites,^35^ while the only two reports on double/triple-cations and double-halide perovskites by SSNMR concern MA_0.15_FA_0.85_PbI_2.55_Br_0.45_^33^ and Cs_0.05_MA_0.16_FA_0.79_PbI_2.49_Br_0.51_.^36^

In this article, we applied for the first time multinuclear—^133^Cs, ^13^C, ^1^H, ^207^Pb—SSNMR experiments for the characterization of the triple-cation lead mixed-halide perovskite with formula Cs_0.05_MA_0.14_FA_0.81_PbI_2.55_Br_0.45_ (in the following indicated as TripleMix), which was found to be in one of the most efficient stoichiometries for single-junction solar cells.^7^ In this SSNMR investigation, we exploited several nuclear probes, complementing spectroscopy experiments with the measurement of nuclear relaxation times, with the aim of highlighting structural and dynamic features related to TripleMix specific stoichiometry.

TripleMix was prepared following the procedure described in the Supporting Information, based on a previously reported synthesis.^37^ Powder X-ray diffraction (PXRD, Figure S1) confirmed the obtainment of a single perovskite tetragonal phase. MAPbI_3_ and MAPbBr_3_ parent compounds were also prepared (see Supporting Information) with the aim of analyzing them by means of ^207^Pb SSNMR and comparing the results with those of TripleMix. Optical characterization, including UV–vis absorption, PL, and Time-Resolved Photoluminescence (TRPL) measurements, was performed on thin films typically used in devices (conditions are detailed in Supporting Information); the results are shown in Figure 1b. MAPbI_3_ thin films have a band gap in the near-infrared spectrum, as shown by the onset of the UV–vis absorption at 780 nm. PL is resonant with the band gap, with very limited Stokes shift and few tens of nanometers in width. The trend of PL under femtosecond pulsed excitation, measured with a streak camera, shows a characteristic decay time of several nanoseconds, limited by defects and traps. The time decays become progressively shorter for growing laser fluences, due to the bimolecular nature of carrier recombination in perovskites.^38^ TripleMix films show very similar optical properties, with a longer PL decay time, due to the reduced nonradiative recombination rate.^39^ MAPbBr_3_ films, instead, have a band gap significantly blue-shifted, due to the effect of the smaller halide radius.

Insights into the structure and dynamics of TripleMix were obtained by SSNMR looking at all present cations through NMR-active nuclei. Starting from A^+^ cations, ^133^Cs SSNMR can give information on cesium doping ions, known to play a crucial role in perovskite performance and stability. ^133^Cs is a quadrupolar nucleus (*I* = 7/2), and its high NMR receptivity allows experiments to be carried out even on samples with a very small amount of cesium, like TripleMix. Moreover, the chemical shift of ^133^Cs (δ(^133^Cs)) is highly sensitive to its local environment. The ^133^Cs Magic Angle Spinning (MAS) spectrum of TripleMix (Figure 2a) shows a single signal centered at ca. 37 ppm, with a Full Width at Half-Maximum (fwhm) of about 1.5 kHz. δ(^133^Cs), reported in the literature for different perovskites (Table 1), shows a remarkable sensitivity to tiny variations in sample composition. Indeed, in single-cation single-anion perovskites, by changing the halogen, δ(^133^Cs) varies from 77 ppm in CsPbCl_3_ to 110 and 167 ppm in CsPbBr_3_ and γ-CsPbI_3_, respectively.^40^ When cesium is introduced in hybrid perovskites, δ(^133^Cs) strongly decreases: values from 26 to 50 ppm are reported for δ(^133^Cs) in Cs_*x*_FA_1–*x*_PbI_3_ with *x* ranging from 0.1 to 0.3.^35^ The value of 37 ppm, here measured for TripleMix, allows us to rule out the presence of CsPbX_3_ phases and to assess the full incorporation of Cs^+^ in the mixed perovskite phase. It is interesting to observe that δ(^133^Cs) is also sensitive to the presence and amount of MA, as evinced by comparing the value determined for TripleMix with those, quite different, found for Cs_*x*_FA_1–*x*_PbI_3_ and that of 44 ppm recently reported for a perovskite with chemical formula Cs_0.05_MA_0.16_FA_0.79_PbI_2.49_Br_0.51_.^36^ The line width of the ^133^Cs signal in the MAS spectrum of TripleMix (fwhm ≈ 1.5 kHz) is similar to that observed for the other mixed perovskites,^34−36^ while in the spectra of pure CsPbBr_3_ and CsPbI_3_ phases the ^133^Cs signal is much narrower (fwhm ≈ 200–400 Hz).^40^ This finding suggests that, in the mixed samples, a distribution of shifts can arise from slightly different ^133^Cs environments. Moreover, a reduction of symmetry with respect to that of pure cubic phases can occur in the Cs environment in mixed cation and anion samples, and the line width can consequently be broadened due to quadrupolar coupling. We also recorded ^133^Cs Rotor-synchronized Hahn-echo experiments at different MAS frequencies (Figure S3b,c), which showed spinning sidebands, in agreement with the static spectrum (Figure S3a).

**Figure 2:**
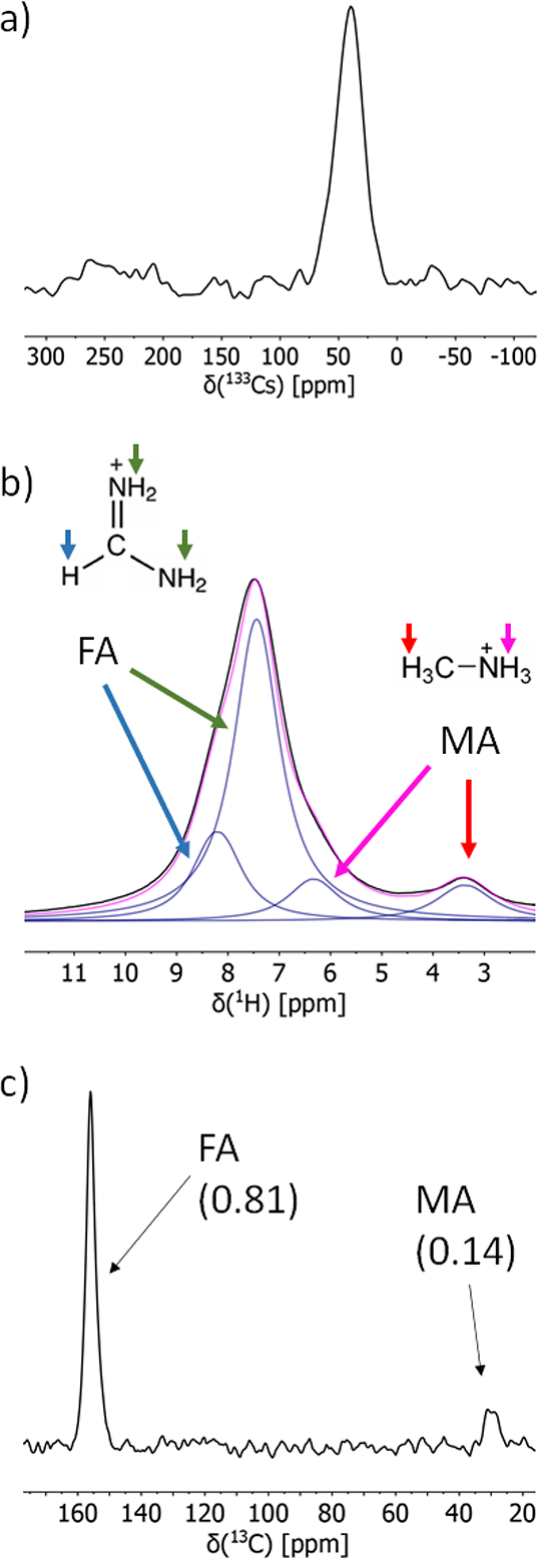
^133^Cs (a), ^1^H (b), and ^13^C (c) MAS NMR spectra of TripleMix. (b, c) Signal assignments to MA and FA hydrogen and carbon nuclei are also indicated. (c) The relative molar amounts of MA and FA expected for TripleMix are shown in parentheses.

**Table 1:** ^133^Cs Chemical Shift Values, δ(^133^Cs), of TripleMix and Other Cs-Containing Perovskites Reported in the Literature^35,36,40^

perovskite	δ(^133^Cs), ppm
Cs_0.05_MA_0.14_FA_0.81_PbI_2.55_Br_0.45_ (TripleMix this work)	37
Cs_0.05_MA_0.16_FA_0.79_PbI_2.49_Br_0.51_	∼44^36^^a^
CsPbBr_3_	110^40^
γ-CsPbI_3_	167^40^
Cs_*x*_FA_1–*x*_PbI_3_	
(*x* = 0.10)	26,^35^^a^
(*x* = 0.15)	31
(*x* = 0.20)	39
(*x* = 0.30)	50

a These δ(^133^Cs) values were shifted by +13 ppm with respect to those reported in refs (35) and (36) to be consistent with the IUPAC convention here used for the ^133^Cs reference.

As far as organic cations are concerned, ^1^H and ^13^C MAS spectra of TripleMix (Figure 2b and c) show the signals of MA and FA and allow a quantification of the FA:MA ratio. This information is not easily obtainable from other techniques, and ^1^H SSNMR has been previously proposed as a method of choice for measuring the FA:MA ratio.^28^ The ^1^H MAS spectrum shows two quite broad peaks: a weak one centered at about 3.4 ppm and one, much more intense, centered at about 7.5 ppm. The peak at 3.4 ppm can be straightforwardly ascribed to the sole MA methyl protons, whereas the peak at 7.5 ppm must include the signals of the remaining ^1^H nuclei from both MA and FA cations. This becomes clearer by performing a spectral fitting (Figure 2b), which reveals that the signal at higher chemical shift is indeed constituted by three signals at 6.3, 7.4, and 8.2 ppm, which can be assigned to NH_3_ of MA, and NH_2_ and CH of FA, respectively. The chemical shift values are in agreement with those reported for pure parent perovskites (MAPbI_3_, FAPbI_3_) and it has been already reported that they do not significantly change in double-cation compositions^32,33^ and in a triple-cation double-halide perovskite with stoichiometry slightly different from that of TripleMix.^36^ The signal areas found by spectral fitting were used to quantify the FA:MA ratio, obtaining a ratio of 0.80:0.15 mol:mol, in good agreement with that expected for TripleMix based on the precursors’ (MABr and FAI) stoichiometry used in the synthesis (0.81:0.14 mol:mol).

The ^13^C Direct Excitation (DE) MAS spectrum of TripleMix (Figure 2c) shows one intense signal at 156.0 ppm arising from the CH group of FA and one weak signal at 29.9 ppm for the CH_3_ group of MA. The observed chemical shifts are quite similar to those reported for mixed MA/FAPbI_3_ perovskites,^32^ indicating that they are not affected by the presence of Cs^+^ and Br^–^. Since the spectrum is recorded in quantitative conditions, ensured by a recycle delay between consecutive transients of 75 s, it is possible to obtain a second check for the FA:MA ratio (Figure 2c), which results to be 0.81:0.14 mol:mol.

^1^H nuclei can be excellent probes also to investigate the dynamics of FA and MA ions, which has been shown to be related to PV performance.^41−44^ To this aim, proton longitudinal relaxation times, T_1_(^1^H), were measured at room temperature (RT) at two different Larmor frequencies on TripleMix; the obtained values are reported in Table 2, together with literature data on different perovskites. A single T_1_ value is measured at both frequencies, as also reported for other mixed cation perovskites.^32,33^ The fact that all ^1^H nuclei show a single T_1_ value is a further evidence that a single phase forms, without segregation of different phases. Indeed, if MA and FA would separate in different phases, two different T_1_’s could be expected (see Table 2 for T_1_(^1^H) values of parent perovskites). On the contrary, if organic cations are intimately mixed in a single perovskite phase, spin-diffusion, a diffusion of longitudinal magnetization occurring through homonuclear ^1^H–^1^H dipolar couplings, averages different intrinsic T_1_’s to a single value.^45^

**Table 2:** T_1_(^1^H) Values of TripleMix and Other Perovskites (Taken from the Literature) Measured at Room Temperature

perovskite	^1^H Larmor frequency	T_1_(^1^H), s
TripleMix (this work)	400 MHz	16 ± 0.5
	500 MHz	18 ± 0.5
MAPbBr_3_	400 MHz	18^46^
	20 MHz	ca. 18^47,48^
MAPbI_3_	400 MHz	16^49^
	400 MHz	15^50^
	20 MHz	14^51^
FA_0.67_MA_0.33_PbI_3_	500 MHz	26^32^
FAPbBr_3_	700 MHz	ca. 29^34^
Cs_0.05_FA_0.95_PbBr_3_	700 MHz	ca. 23^34^
α-FAPbI_3_	500 MHz	33^48^

The dynamics of organic cations in LHPs has been extensively investigated by SSNMR. Early studies on MAPbX_3_, with X^–^ = Cl^–^, Br^–^, I^–^, via ^1^H, ^2^H, and ^14^N SSNMR spectroscopy,^47,52,53^ showed that MA cations are subjected to a rapid reorientation of the C–N axis at RT, in addition to a very fast rotation along the C–N axis. In recent years, the interest in perovskites for solar cell applications drove a new series of SSNMR studies, which confirmed that, in these perovskites, MA cations undergo a fast jump-like reorientation of the C–N axis at RT.^49^ In the case of MAPbI_3_, a line-shape analysis of ^2^H and ^14^N NMR spectra also allowed the motion geometry to be described in detail and the correlation times to be determined.^54^ Fabini et al. investigated the dynamics of the FA cation in FAPbI_3_, concluding that its correlation time at RT is comparable to that of MA but the activation energy is much lower.^48^ Kubicki et al. used variable-temperature ^1^H, ^2^H, ^13^C, and ^14^N SSNMR spectroscopy to elucidate the dynamics of the MA and FA cations in pure (MAPbI_3_ and FAPbI_3_) and mixed cation (FA_0.67_MA_0.33_PbI_3_) perovskites. They concluded that FA reorients faster than MA at RT, but they found no significant differences in the dynamics of both MA and FA in the mixed cation perovskite with respect to the pure reference compounds.^32^ In addition, Mozur et al. investigated the FA dynamics in Cs-doped FAPbBr_3_, concluding that the crystal distortion introduced by doping disrupts the concerted motions of FA cations at low temperature.^34^

With respect to the perovskites studied in the previously cited literature, TripleMix represents a step forward in the composition complexity, and this is the first time in which T_1_(^1^H) values are reported for MA and FA coexisting with Cs, I, and Br in the same lead perovskite. Although a full characterization of the dynamics of organic cations in TripleMix is out of the scope of this work, T_1_(^1^H) values measured at RT give us some hints. T_1_(^1^H) values reported for several parent perovskites (Table 2) turned out to be independent of the Larmor frequency at RT or above. From a first comparison, we can notice that T_1_(^1^H) of TripleMix is very similar to the lowest values reported in Table 2, thus suggesting that it cannot represent an average of the T_1_(^1^H) values of the parent compounds. To verify this hypothesis, we calculate an average relaxation time as the inverse of the Population Weighted Relaxation Average (PWRA),^55,56^ expressed by the following equation
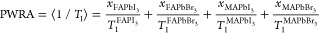
under the assumption that the intrinsic T_1_(^1^H) of MA and FA does not change when passing from parent compounds to TripleMix and neglecting the possible effect of Cs^+^. Using T_1_ values reported in Table 2 for the pure parent perovskites and calculating the proton molar fractions (*x*_i_) from the general formula of TripleMix, we obtain 1/PWRA = 26 s. Even considering all the approximations, the calculated value is quite larger than the experimental one, thus suggesting a change in the dynamics of organic cations in TripleMix with respect to parent compounds FAPbI_3_, FAPbBr_3_, MAPbI_3_, and MAPbBr_3_. This effect, not observed in the case of the mixed sample FA_0.67_MA_0.33_PbI_3_,^32^ could be related to the presence of a small amount of Cs^+^, as reported for FA in Cs_*x*_FA_1–*x*_PbBr_3_.^34^ In particular, Cs^+^ could induce distortions in the octahedra cavities, which could result in a slowdown of the dynamics of FA and MA. This effect was indeed found by Ghosh et al.^57^ in Cs_*x*_FA_1–*x*_PbI_3_ through ab initio simulations. The computational study showed that the incorporation of Cs^+^ cations induces significant structural distortions and that motions of FA cations are significantly inhibited in the mixed samples with respect to FAPbI_3_.

To obtain information on the inorganic framework of TripleMix we exploited the SSNMR observation of ^207^Pb nuclei. Interesting information was obtained from the ^207^Pb static spectrum of TripleMix, which shows a single very broad signal centered at about 1490 ppm, with fwhm of 34.9 kHz (Figure 3). It is worth noticing that no other signals are observed (even in spectra registered with frequency-stepped acquisition^58^), which confirms the formation of a single phase and excludes the presence of pure parent perovskites and/or PbI_2_ that may arise from degradation.

**Figure 3:**
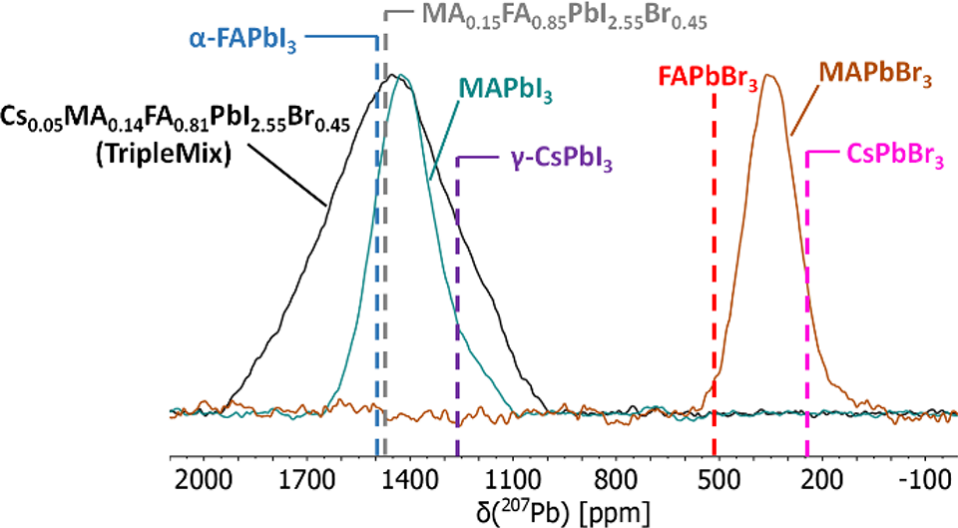
Static ^207^Pb SSNMR spectra of TripleMix (black) and of the parent perovskites MAPbI_3_ (green) and MAPbBr_3_ (brown). Dashed lines indicate ^207^Pb chemical shifts in α-FAPbI_3_ (blue), FAPbBr_3_ (red), γ-CsPbI_3_ (purple), CsPbBr_3_ (pink), and MA_0.15_FA_0.85_PbI_2.55_Br_0.45_ (gray) taken from the literature^31,33,59^ (see Table 3).

Literature data on APbX_3_ perovskites (see Table 3) indicate that both the halide and the A^+^ cation affect the ^207^Pb chemical shift, δ(^207^Pb), but the effect of the halide is much stronger. Indeed, in passing from APbBr_3_ to APbI_3_ a shift of about +1000 ppm is observed.^31^ On the other hand, δ(^207^Pb) of α-FAPbI_3_ > δ(^207^Pb) of MAPbI_3_ > δ(^207^Pb) of γ-CsPbI_3_ with a maximum variation of about 230 ppm. Rosales et al.^60^ demonstrated a linear dependence of δ(^207^Pb) on the molar fraction of halide in MAPbI_(3–*x*)_Br_*x*_. A linear dependence can also be found for the FAPbI_(3–*x*)_Br_*x*_ series (Figure S4), from which a δ(^207^Pb) value of about 1254 ppm can be predicted for FAPbBr_0.45_I_2.55_. Under the hypothesis that the presence of MA and Cs^+^ would further shift the ^207^Pb signal to a lower chemical shift, the chemical shift value here measured for TripleMix indicates that the effect of triple-ion mixing is more complex than the linear effect expected from the sole halogen mixing. Similar findings were already observed, albeit not discussed, by Grüninger et al.,^33^ who reported a slight shift to a higher frequency in passing from MA_0.25_FA_0.75_PbI_3_ to MA_0.15_FA_0.85_PbI_2.55_Br_0.45_. It is also interesting to notice that the presence of a small amount of cesium in TripleMix determines a slight but significant shift of δ(^207^Pb) to lower values with respect to the very similar mixed system MA_0.15_FA_0.85_PbI_2.55_Br_0.45_ (Table 3).^33^

**Table 3:** Chemical Shift, δ, Line Width (fwhm), and T_1_ of ^207^Pb in MAPbBr_3_, MAPbI_3_, and TripleMix (Measured in This Work) Compared with Those of Other Relevant Perovskites Taken from the Literature. All T_1_ Values Were Measured in Static Conditions

perovskite	δ (^207^Pb), ppm	fwhm, kHz	T_1_, s
MAPbBr_**3**_ (this work)	361	14.9	1.54 ± 0.10^a^
			1.54^a^^,^^60^
MAPbI_**3**_ (this work)	1423	17.3	1.32 ± 0.10^a^
			1.07^a^^,^^60^
TripleMix (this work)	1450	34.9	0.75 ± 0.10^a^
CsPbBr_3_	244^40^	13.8^59^	
γ-CsPbI_3_	1265^40^	20^59^	
FAPbBr_3_	510^30,31^	15^31^	1.85^b^^,^^31^
α-FAPbI_3_	1495^30,31^	ca. 22–26^31^	1.94^b^^,^^31^
MA_0.15_FA_0.85_PbI_2.55_Br_0.45_	ca. 1480^33^	ca. 33^33^	
MA_0.25_FA_0.75_PbI_3_	ca. 1460^33^		
MA_0.5_FA_0.5_PbI_3_	ca. 1450^33^		
MA_0.75_FA_0.25_PbI_3_	ca. 1440^33^		

a Measured at a magnetic field of 9.4 T.

b Measured at a magnetic field of 7.0 T.

A noticeable effect of the presence of mixed cations and anions is observed on the line width of the ^207^Pb signal; indeed, the fwhm measured in TripleMix is almost twofold that for MAPbI_3_ and MAPbBr_3_ (Table 3). Generally, ^207^Pb signals are broad also in single-cation single-halide perovskites. This is mainly ascribable to ^207^Pb–X scalar coupling combined with a short spin–spin relaxation time (*T*_2_).^59^^207^ The ^207^Pb spectral line shape has been shown to be very sensitive to the nature of the halide ions, with the line broadening increasing passing from Cl^–^ to I^–^.^31^ By comparison with similar perovskites with mixed cations^33^ and anions,^31^ the line width of the TripleMix ^207^Pb signal can be ascribed to a chemical shift distribution generated by the random distribution of halide ions surrounding each lead nucleus in the coordination octahedra, with an additional contribution from the ^207^Pb-X scalar couplings. Our findings are in agreement with data reported for MA_0.15_FA_0.85_PbI_2.55_Br_0.45_^33^ and can therefore confirm that, in this compositional range, cation/anion mixing has the most prominent effect on the ^207^Pb signal broadening.

Finally, T_1_(^207^Pb) values were measured at RT for TripleMix and parent compounds MAPbI_3_ and MAPbBr_3_ to get insight into the dynamics of [PbX_6_]^4–^ octahedra. Under MAS conditions, the spin–lattice relaxation of ^207^Pb nuclei is strongly enhanced by a MAS-induced polarization exchange between halogens and lead nuclei,^50,60−62^ with a reduction of T_1_ by more than 1 order of magnitude, which prevents other dynamic information to be obtained. Here, T_1_(^207^Pb) values were measured by saturation recovery experiments combined with the Hahn-echo sequence in static conditions. For all samples, the recovery curve was well-reproduced by a single exponential function; the determined T_1_(^207^Pb) values are reported in Table 3, together with values for FAPbBr_3_ and α-FAPbI_3_ taken from the literature. By comparing T_1_ data, an enhanced ^207^Pb relaxation is evident in TripleMix with respect to MA- and FA-based parent compounds.

^207^Pb spin–lattice relaxation is complicated, and no interpretation of T_1_(^207^Pb) values has been reported in the literature for LHPs. In a study on PbI_2_, Taylor et al.^63^ measured T_1_(^207^Pb) at variable temperature and hypothesized two possible physical models for the interpretation: a Raman-like mechanism or a thermally activated mechanism. The first is a lattice-vibration-based relaxation mechanism previously reported for heavy spin-1/2 nuclei,^64^ which implies a peculiar dependence of T_1_ on *T* (T_1_ ∝ *T*^–2^) and no magnetic field dependence. On the other hand, the most common thermally activated mechanism assumes that there is a correlation time that characterizes the modulation of the local magnetic interactions in the surrounding of the ^207^Pb nuclei. In the case of PbI_2_, the authors supposed that the thermally activated process could be iodine ion hopping (observed by ^127^I T_1_), which affects ^207^Pb relaxation through scalar coupling, but this hypothesis has not been confirmed yet.

Motions of the octahedra in LHPs have been postulated and observed by SSNMR, but a complete understanding is still missing. In particular, from variable-temperature ^207^Pb *T*_2_ measurements Kentgens and colleagues^65^ concluded that [PbI_6_]^4–^ octahedra in MAPbI_3_ are subjected to a slow motion. In addition, Aebli et al. were able to resolve Pb–Br scalar couplings in low-temperature ^207^Pb SSNMR spectra and ascribed the lower resolution at RT to structural dynamics and/or a degree of disorder in APbBr_3_ perovskites (with A^+^ = Cs^+^, MA, and FA).^59^ Molecular dynamics simulations of Cs_*x*_FA_1–*x*_PbI_3_ showed that dynamic tilting of [PbI_6_]^4–^ octahedra is inhibited by Cs^+^ incorporation.^57^ In addition, halide ion migration has been shown to be slower in Cs_0.3_MA_0.7_PbBr_1.5_I_1.5_ and Cs_0.5_MA_0.5_PbBr_1.5_I_1.5_ than in MAPbBr_1.5_I_1.5_.^66,67^ The lower value of T_1_(^207^Pb) measured in this work for TripleMix with respect to pure parent compounds is in agreement with these last results and indicates that dynamic tilting of [PbX_6_]^4–^ octahedra and/or halide mobility are slowed down also in our perovskite.

In conclusion, this is the first multinuclear SSNMR investigation of the very well-performing triple-cation lead double-halide perovskite Cs_0.05_MA_0.14_FA_0.81_PbI_2.55_Br_0.45_, including both recording of spectra and measurements of nuclear spin–lattice relaxation times. From a detailed comparison with data on parent compounds, it emerged that the chemical shift of ^207^Pb in TripleMix is intermediate between those of FAPbI_3_ and MAPbI_3_, while the presence of a small amount of bromine does not lead to a significant decrease of chemical shift, in agreement with data reported for MA_0.15_FA_0.85_PbI_2.55_Br_0.45_.^33^ Moreover, the comparison with MA_0.15_FA_0.85_PbI_2.55_Br_0.45_ highlighted a decrease of the chemical shift ascribable to the presence of the small amount of cesium. The ^207^Pb signal line width, larger than that of pure LHPs and comparable to that of MA_0.15_FA_0.85_PbI_2.55_Br_0.45_, indicated a distribution of Pb coordination environments. Interestingly, the T_1_ of ^207^Pb nuclei was noticeably lower than those of parent pure compounds, suggesting a peculiarly enhanced relaxation related to the mixed composition.

As far as A^+^ cations are concerned, ^1^H, ^13^C, and ^133^Cs NMR spectra confirmed the stoichiometry of TripleMix and the effective mixing of the cations, ruling out the occurrence of phase segregation. Similarly to what observed for ^207^Pb, the ^1^H nuclei of the organic cations showed an enhanced spin–lattice relaxation. Indeed, T_1_(^1^H) values indicated interesting differences in the dynamics of organic cations with respect to pure compounds, suggesting that the mixing induces structural changes that could slow down the reorientational motion of FA and MA, not observed for other mixed-cation perovskites. These results suggest an important role of Cs^+^ in altering the dynamic properties of MA and FA, which could be related to the suppression of collective organic and inorganic motions, in turn affecting carrier lifetimes. This hypothesis is supported by a previous computational study^57^ and by the observation of the effect of Cs^+^ in altering the concerted cation dynamics in Cs_*x*_FA_1–*x*_PbBr_3_,^34^ while the correlation between altered dynamics of FA/MA and changes in carrier lifetimes was highlighted by studying the effect of deuteration in MAPbI_3_ and FAPbI_3_.^41−43^

The results obtained in this work encourage us to further investigate the dynamics of cations in complex perovskites by measuring nuclear relaxation times at variable temperature and magnetic field.

## Supporting Information Available

Experimental details, materials, and methods; additional structural and optical properties of TripleMix and parent perovskites; additional ^133^Cs spectra and details about ^207^Pb chemical shift analysis (PDF)

## Abbreviations

DE: direct excitation
FA: formamidinium
fwhm: full width at half-maximum
LHP: lead halide perovskite
MA: methylammonium
MAS: magic angle spinning
PL: photoluminescence
PSC: perovskite solar cells
PV: photovoltaic
PXRD: powder X-ray diffraction
RT: room temperature
SSNMR: solid state nuclear magnetic resonance
TRPL: time-resolved photoluminescence
