# Supplementary material for: Solid-State Nuclear Magnetic Resonance of Triple-Cation Mixed-Halide Perovskites
Source: J Phys Chem Lett. 2022 Oct 6;13(40):9517–25. doi: 10.1021/acs.jpclett.2c02313 (PMC9575147; doi:10.1021/acs.jpclett.2c02313)
Supplement: Supplementary file 1 — jz2c02313_si_001.pdf [file jz2c02313_si_001.pdf]

Supporting Information

Solid State Nuclear Magnetic Resonance of Triple-Cation Mixed-Halide Perovskites

Noemi Landi[1], Elena Maurina[1,§], Daniela Marongiu[2], Angelica Simbula[2], Silvia Borsacchi[3,4], Lucia Calucci[3,4], Michele Saba[2], Elisa Carignani*[3], Marco Geppi[1,3,4]*

[1] Department of Chemistry and Industrial Chemistry, University of Pisa, via G. Moruzzi 13, 56124 Pisa, Italy;

[2] Department of Physics, University of Cagliari, S.P. Monserrato-Sestu Km. 0700, Monserrato, 09042 Cagliari, Italy.

[3] Institute for the Chemistry of OrganoMetallic Compounds - ICCOM, Italian National Research Council - CNR, via G. Moruzzi 1, 56124 Pisa, Italy;

[4] Center for Instrument Sharing, University of Pisa (CISUP), 56126 Pisa, Italy;

[§]Current address: Information Engineering Department, University of Pisa, via G. Caruso 16, 56122, Pisa - Italy

S1. Experimental Section

Perovskites' synthesis. Thin films of TripleMix ($Cs_{0.05}MA_{0.14}FA_{0.81}PbI_{2.55}Br_{0.45}$) were deposited by a two-step spin coating procedure assisted by an antisolvent.[1] A 1.05 M solution of $Cs_{0.05}MA_{0.14}FA_{0.81}PbI_{2.55}Br_{0.45}$ was prepared by dissolving stoichiometric amounts of PbI_2, $PbBr_2$, FAI, MABr and CsI in a mixed Dimethylformamide / Dimethyl sulfoxide solvent (DMF:DMSO = 4:1 vol:vol). A few drops of solution were spun at 2000 rpm for 10 s then at 4000 rpm for 20 s. Chlorobenzene (10 μl) was dropped on the film ten seconds before the end of spinning as antisolvent to help the crystallization process. The film was then annealed at 100 °C on a hot plate. Powders for powder X-ray diffraction (PXRD) and solid state nuclear magnetic resonance (SSNMR) measurements were obtained by removing thin films deposited on glass slides with a spatula.

Single crystals of $MAPbI_3$ and $MAPbBr_3$ were grown by the inverse temperature crystallization method[2] starting from a solution of precursors in γ-butyrolactone (GBL) and DMF, respectively. For $MAPbI_3$ crystal growth, a 1.2 M solution of MAI and PbI_2 in GBL was heated at 90 °C until the crystal stopped growing, while for $MAPbBr_3$ a 1 M solution of MABr and $PbBr_2$ in DMF was heated at 60 °C. Both the crystals were washed with isopropanol and dried before grinding in a mortar. Thin films of $MAPbI_3$ for optical measurements were deposited by spin coating at 6000 rpm a 0.5 M solution of PbI_2 in DMF for 30 s on a glass slide followed by the casting of some drops of a MAI solution in isopropanol (10 mg/ml). The films were then annealed at 100 °C for 1 h and washed with isopropanol. The $MAPbBr_3$ films were deposited by spin coating a solution of MABr and $PbAc_2 \times 3H_2O$ with a 3:1 molar ratio in DMF at 6000 rpm for 60 s on a glass slide followed by thermal annealing at 100 °C for 10 min. All the sample preparations were fulfilled

inside a nitrogen-filled glove box. Powders for PXRD and SSNMR measurements were obtained by grinding single crystals.

All the solvents and inorganic salts were purchased from Meck, while organic salts were purchased from Greatcell Solar and used without further purification.

X-ray diffraction measurements. PXRD measurements were performed on thin films and ground crystals by a Bruker D8 Advance diffractometer with Cu Kα radiation equipped with a position sensitive detector (PSD) detector. Patterns were recorded from 10° to 50° with 0.03° step size and 0.2 s time per step.

Optical measurements. Optical transmission and reflection spectra of thin films in the UV-Vis range were measured with a dual-beam spectrophotometer equipped with an integrating sphere accessory (Agilent Technologies Cary 5000 UV-Vis-NIR) in hemispherical configuration.

SSNMR measurements. ^{1}H and ^{133}Cs SSNMR spectra were collected on a 11.7 T Bruker Avance Neo 500 spectrometer, working at Larmor Frequencies of 500.13 and 65.60 MHz for ^{1}H and ^{133}Cs, respectively. A 2.5 mm cross polarization-magic angle spinning (CP-MAS) probe was used with π/2 pulse duration of 2.3 μs and 10 μs for ^{1}H and ^{133}Cs, respectively. The chemical shift for all nuclei was referenced to the ^{13}C signal of adamantane at 38.46 ppm.

^{1}H MAS spectra were acquired by direct excitation (DE). Longitudinal relaxation times, T_1, of ^{1}H nuclei were measured with the inversion recovery pulse sequence under MAS. 26 delays ranging from 1 ms to 150 s were used, accumulating 4 transients.

^{133}Cs spectra were acquired using the Hahn-echo pulse sequence, under MAS and static conditions, accumulating 400 scans and 240 scans, respectively. In order to optimize acquisition conditions, echo delays ranged from 20 to 66.7 μs, under rotor-synchronized and not synchronized conditions.
^{1}H, ^{13}C, and ^{207}Pb SSNMR spectra were collected on a 9.4 T Varian Infinity Plus 400 spectrometer, working at Larmor Frequencies of 400.34, 100.67, and 83.53 MHz for ^{1}H, ^{13}C, and ^{207}Pb, respectively, by using a 3.2 mm CP-MAS probe with $\pi/2$ pulse duration of 2.3 μs, 5.8 μs, and 5 μs for ^{1}H, ^{13}C, and ^{207}Pb, respectively. The chemical shift was referenced to the signal of adamantane at 1.7 ppm for ^{1}H, to the signal of adamantane at 38.46 ppm for ^{13}C and to that of lead nitrate (-3490 ppm) for ^{207}Pb, as secondary references.
^{1}H T_1 values were measured with the inversion recovery pulse sequence under MAS. 28 delays ranging from 1 ms to 80 s were employed, accumulating 8 transients.

^{13}C MAS spectra were acquired by direct excitation with background suppression (DEPTH sequence[3]) under high-power proton decoupling using a recycle delay of 75 s (optimized to guarantee quantitative spectra by means of preliminary experiments) and accumulating 10000 transients.
^{207}Pb spectra were acquired using the Hahn-echo pulse sequence under static and MAS conditions, with an echo delay of 12.5 μs and accumulating at least 80000 transients. MAS conditions (up to 15 kHz) did not significantly affect the ^{207}Pb line width of our samples. Frequency-stepped acquisition[4] was tested but resulted not necessary for all the investigated samples. T_1 values of ^{207}Pb nuclei were obtained by saturation recovery combined with the Hahn-echo sequence under static conditions. 22 delays ranging from 100 μs to 10 s were used, accumulating 5000 transients.
All MAS experiments were performed at a spinning frequency of 15 kHz unless otherwise stated.
All the experiments were performed at room temperature (298 K).

S2. Structural and optical properties of TripleMix and parent perovskites

All the powders were analyzed by PXRD to confirm the crystallographic phase and the presence of a single phase. Optical characterization such as UV-Vis absorption, photoluminescence (PL) and time-resolved photoluminescence (TRPL) were performed on thin films typically used in devices.

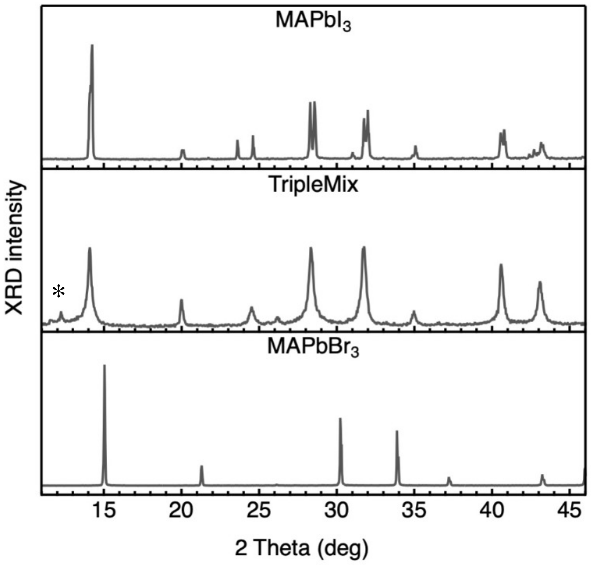

Figure S1. PXRD patterns of $MAPbI_3$, TripleMix and $MAPbBr_3$. The asterisk indicates a small amount of PbI_2.

PXRD patterns show, as expected, a cubic phase for $MAPbBr_3$ and a tetragonal phase for both $MAPbI_3$ and TripleMix. In particular, in the TripleMix pattern a weak peak ascribed to PbI_2 is visible at 12.2°. The presence of PbI_2 is related to the beginning of a decomposition process in the

sample during the measurement in ambient air. To confirm the single phase of TripleMix, XRD on the original thin film was performed in ambient air and no sign of degradation was visible.

Fig. S2 shows the as-deposited TripleMix film before scratching it off the glass slide. It is possible to notice a preferred orientation and the lack of PbI_2 phase, confirming the purity of the material used for SSNMR measurements.

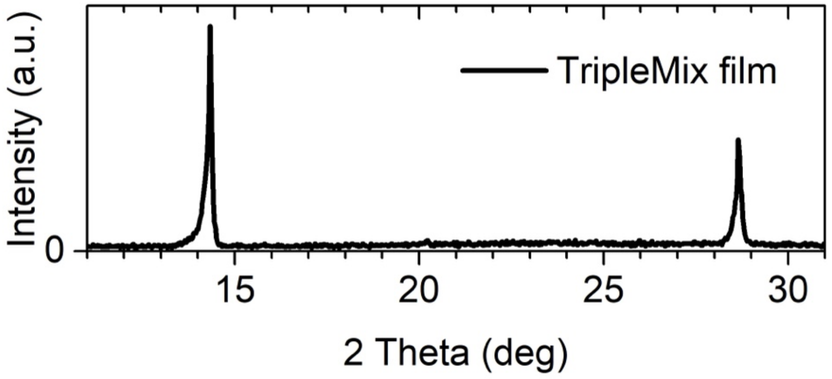

Figure S2. PXRD pattern of the original TripleMix thin film. No decomposition is visible.

S3. ^{133}Cs SSNMR spectra of TripleMix

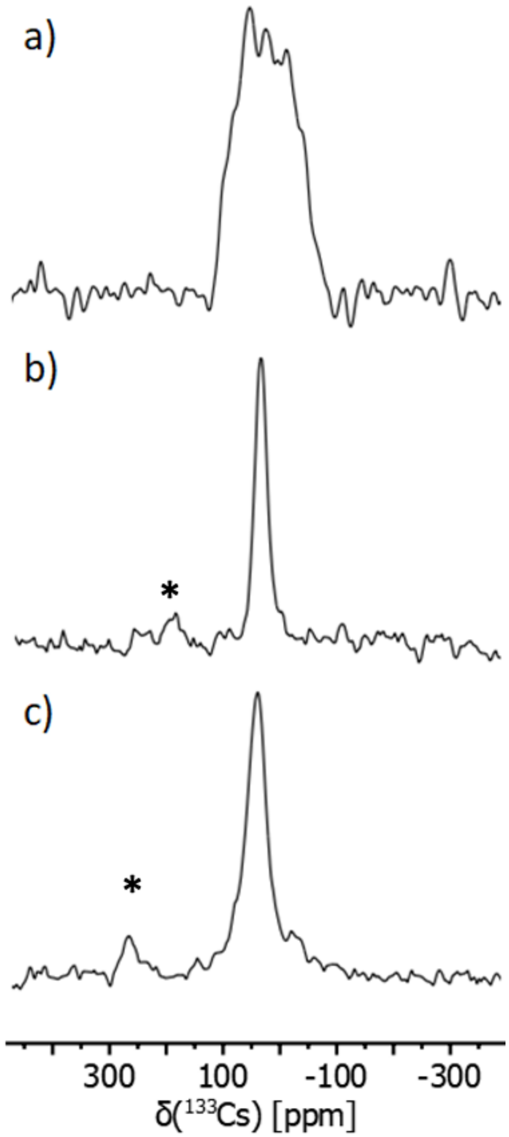

Figure S3. ^{133}Cs SSNMR spectra of TripleMix under static (a) and MAS at 10 kHz (b) and 15 kHz (c). Spectra in (b) and (c) have been acquired using the Hanh-echo pulse sequence with the echo delays synchronized with MAS. Spinning sidebands are marked with asterisks.

^{133}Cs MAS spectra recorded with rotor-synchronized echoes show the presence of a spinning sideband (marked with asterisk in Figure S3) both at 15 and 10 kHz, which is not present in the spectrum recorded with shorter – not rotor synchronized – echo delay reported in the main text.

S4. ^{207}Pb chemical shift

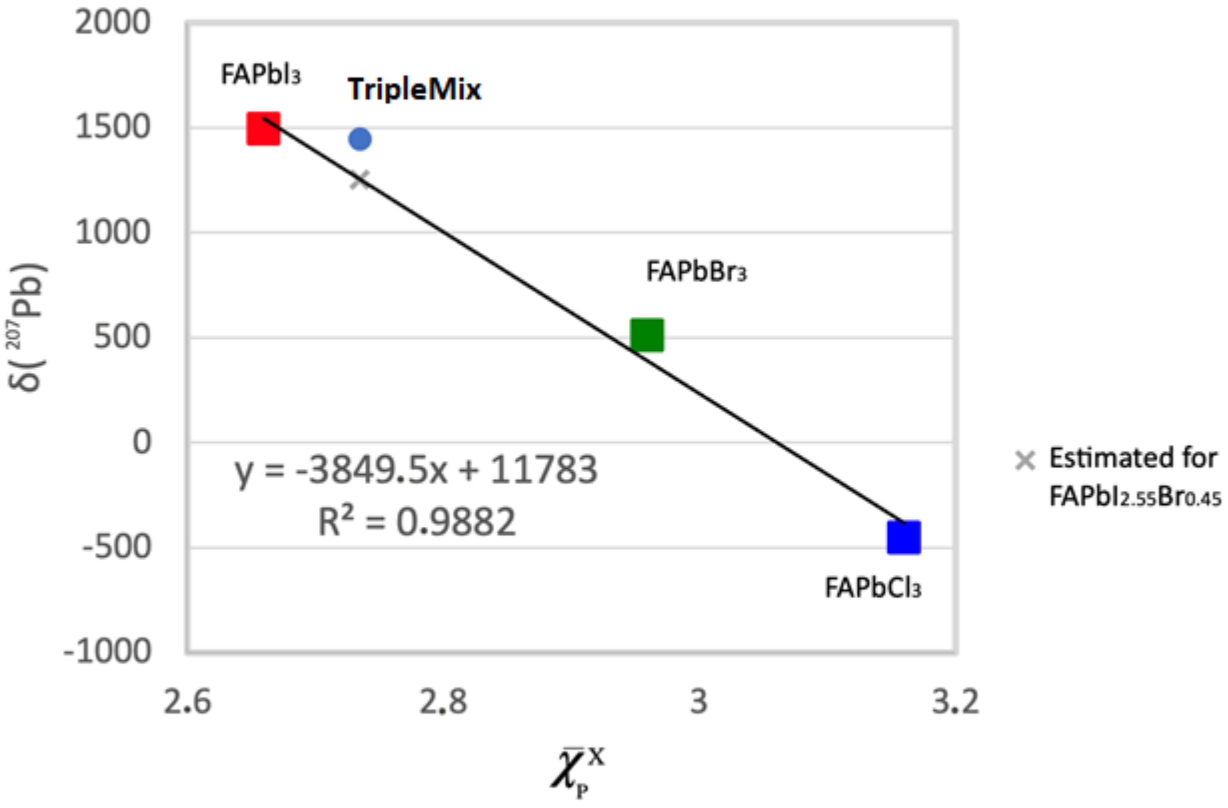

Figure S4. Trend of δ(^{207}Pb), reported in ppm, as a function of halogens electronegativity ($\bar{\chi}_P^X$), experimental δ(^{207}Pb) measured for TripleMixed (blue circle), and δ(^{207}Pb) estimated for the mixed $FAPbI_{2.55}Br_{0.45}$ (gray cross), by using the equation reported on graph. $\bar{\chi}_P^X$ values are taken from ref 5. δ(^{207}Pb) values for $FAPbX_3$ are taken from ref 6.

Following the procedure proposed by Rosales et al.[7], a linear dependence of δ(^{207}Pb) on the Pauli's electronegativity $\bar{\chi}_P^X$ of the halogens can be observed for the $FAPbX_3$ series (Fig. S4). The linear fit of δ(^{207}Pb) in $FAPbCl_3$, $FAPbBr_3$ and $FAPbI_3$ *vs.* $\bar{\chi}_P^X$ yields:

$$\delta(^{207}\mathrm{Pb}) = 11783 - 3849.5\ \bar{\chi}_P^X$$

Using this equation a δ(^{207}Pb) value of 1254 ppm is estimated for the mixed $FAPbI_{2.55}Br_{0.45}$ perovskite, showing the same stoichiometric I:Br ratio as TripleMix; this value is significantly lower than the 1450 ppm value measured for TripleMix in this work. Considering that, as discussed in the main text (Table 3), the presence of MA and Cs^+ cations in FA-based perovskites has in principle the effect of lowering δ(^{207}Pb), the expected chemical shift should be even lower than the experimental one.[8]

REFERENCES

(1) Tan, H.; Jain, A.; Voznyy, O.; Lan, X.; García de Arquer, F. P.; Fan, J. Z.; Quintero-Bermudez, R.; Yuan, M.; Zhang, B.; Zhao, Y.; Fan, F.; Li, P.; Quan, L. N.; Zhao, Y.; Lu, Z. H.; Yang, Z.; Hoogland, S.; Sargent, E. H. Efficient and Stable Solution-Processed Planar Perovskite Solar Cells via Contact Passivation. *Science* **2017**, *355*, 722–726.

(2) Saidaminov, M. I.; Abdelhady, A. L.; Murali, B.; Alarousu, E.; Burlakov, V. M.; Peng, W.; Dursun, I.; Wang, L.; He, Y.; Maculan, G.; Goriely, A.; Wu, T.; Mohammed, O. F.; Bakr, O. M. High-Quality Bulk Hybrid Perovskite Single Crystals within Minutes by Inverse Temperature Crystallization. *Nat. Commun.* **2015**, *6*, 7586.

(3) Cory, D. G.; Ritchey, W. M. Suppression of Signals from the Probe in Bloch Decay Spectra. *J. Magn. Reson.* **1988**, *80*, 128–132.

(4) Pell, A. J.; Clément, R. J.; Grey, C. P.; Emsley, L.; Pintacuda, G. Frequency-Stepped Acquisition in Nuclear Magnetic Resonance Spectroscopy under Magic Angle Spinning. *J. Chem. Phys.* **2013**, *138*, 114201.

(5) Allred, A. L. Electronegativity Values from Thermochemical Data. *J. Inorg. Nucl. Chem.* **1961**, *17*, 215–221.

(6) Askar, A. M.; Karmakar, A.; Bernard, G. M.; Ha, M.; Terskikh, V. V.; Wiltshire, B. D.; Patel, S.; Fleet, J.; Shankar, K.; Michaelis, V. K. Composition-Tunable Formamidinium

Lead Mixed Halide Perovskites via Solvent-Free Mechanochemical Synthesis: Decoding the Pb Environments Using Solid-State NMR Spectroscopy. *J. Phys. Chem. Lett.* **2018**, *9*, 2671–2677.

(7) Rosales, B. A.; Men, L.; Cady, S. D.; Hanrahan, M. P.; Rossini, A. J.; Vela, J. Persistent Dopants and Phase Segregation in Organolead Mixed-Halide Perovskites. *Chem. Mater.* **2016**, *28*, 6848–6859.

(8) Grüninger, H.; Bokdam, M.; Leupold, N.; Tinnemans, P.; Moos, R.; De Wijs, G. A.; Panzer, F.; Kentgens, A. P. M. Microscopic (Dis)Order and Dynamics of Cations in Mixed FA/MA Lead Halide Perovskites. *J. Phys. Chem. C* **2021**, *125*, 1742–1753.
